# The ACE2/Angiotensin-(1–7)/Mas Receptor Axis: Pleiotropic Roles in Cancer

**DOI:** 10.3389/fphys.2017.00276

**Published:** 2017-05-08

**Authors:** Juanjuan Xu, Jinshuo Fan, Feng Wu, Qi Huang, Mengfei Guo, Zhilei Lv, Jieli Han, Limin Duan, Guorong Hu, Lian Chen, Tingting Liao, Wanli Ma, Xiaonan Tao, Yang Jin

**Affiliations:** Key Laboratory of Respiratory Diseases of the Ministry of Health, Department of Respiratory and Critical Care Medicine, Union Hospital, Tongji Medical College, Huazhong University of Science and TechnologyWuhan, China

**Keywords:** renin-angiotensin system (RAS), angiotensin-converting enzyme 2 (ACE2), angiotensin-(1–7), Mas receptor, cancer, drug resistance

## Abstract

Cancer remains one of the most common causes of death and disability and represents a major economic burden in industrialized nations. The renin-angiotensin system (RAS) has been well-recognized as one of the most important regulators of both normal and pathological physiological processes in the brain, kidney, heart, and blood vessels. The activation of the angiotensin-converting enzyme 2/angiotensin-(1–7)/mitochondrial assembly receptor [ACE2/Ang-(1–7)/MasR] axis, which is one component of the RAS, has recently been identified as a critical component of pulmonary systems, gastric mucosa, and cancer. However, the ability of the ACE2/Ang-(1–7)/MasR axis to suppress or promote cancer has not been fully elucidated. In this review, we focus on recent experimental and clinical studies investigating the basic properties, roles, and mechanisms of ACE2, Ang-(1–7), and the MasR, as well as the axis pathway, to provide insights into possible therapeutic strategies for treating cancer that target the ACE2/Ang-(1–7)/MasR axis.

## Introduction

Cancer is a major cause of mortality and is a public health problem in most parts of the world. Although therapeutic techniques, including chemotherapy, radiotherapy, surgery, and biochemotherapy, have been improved considerably, the death rates associated with cancer remain frustrating (Gallagher et al., 2011; Chen et al., 2016). Therefore, additional therapeutic targets for treating cancer must be developed.

Accumulating evidence indicates that the renin-angiotensin system (RAS) is implicated in the process of cancer (George et al., 2010; Wegman-Ostrosky et al., 2015; Zheng et al., 2015). The classical RAS consists of various axes, including the renin/angiotensin-converting enzyme (ACE)/angiotensin II (Ang II)/Ang II type 1 receptor (AT1R) axis, whose components have been widely identified to play a role in different malignancies, such as ovarian carcinoma (Suganuma et al., 2005), renal cancer (McKay et al., 2015; Zheng et al., 2015), colorectal carcinoma (Neo et al., 2010), and breast cancer (Zhao et al., 2010). Although the classical RAS is considered to play physiological roles in the regulation of cardiovascular and renal function, blood pressure, aldosterone biosynthesis and release, and body salt and fluid balance (Chappell, 2016), imbalances in the RAS may also represent factors that underlie tumor growth, metastasis, and angiogenesis (George et al., 2010). In addition, a newly discovered axis, the angiotensin-converting enzyme 2/angiotensin-(1–7)/mitochondrial assembly receptor [ACE2/Ang-(1–7)/MasR] axis, has been identified, and it acts as a negative regulator of Ang II activity (Donoghue et al., 2000; Santos et al., 2008), whereas AngII induces tumor progression in intrahepatic cholangiocarcinoma (Fyhrquist and Saijonmaa, 2008; Okamoto et al., 2010).

Reports have revealed that ACE2 may have both positive and negative roles in cancer therapies, and it has been identified as an inhibitor of cancer cell growth, metastasis, and angiogenesis in lung cancer (Feng et al., 2010), breast cancer (Yu et al., 2016), colon cancer (Bernardi et al., 2012), and pancreatic cancer (Zhou et al., 2011). Ang-(1–7) has been found to inhibit lung cancer cell growth (Gallagher and Tallant, 2004), but it promotes the migration and invasion of human renal cell carcinoma cells via the Mas-mediated AKT signaling pathway (Zheng et al., 2015), whereas the MasR has been demonstrated to act as an anti-tumor agent in breast cancer (Luo et al., 2015). Thus, the roles of the ACE2/Ang-(1–7)/MasR axis in cancer are complicated, although studies have regarded ACE2 and AngII as therapeutic drugs against cancer (Gallagher et al., 2014). Although the physiological and pathophysiological roles of ACE2 and AngII are not completely understood, numerous experimental studies have suggested that they have notable protective effects against cancer. Therefore, ACE2, Ang-(1–7), and the MasR might represent new therapeutic targets for treating cancer. In this review, we summarize the evidence from experimental and clinical studies on the effects of ACE2, Ang-(1–7), and Mas in various pathological tumor conditions, and specifically elucidate their complicated effects on cancer.

### Conventional vs. alternate RAS

**Figure 1:**
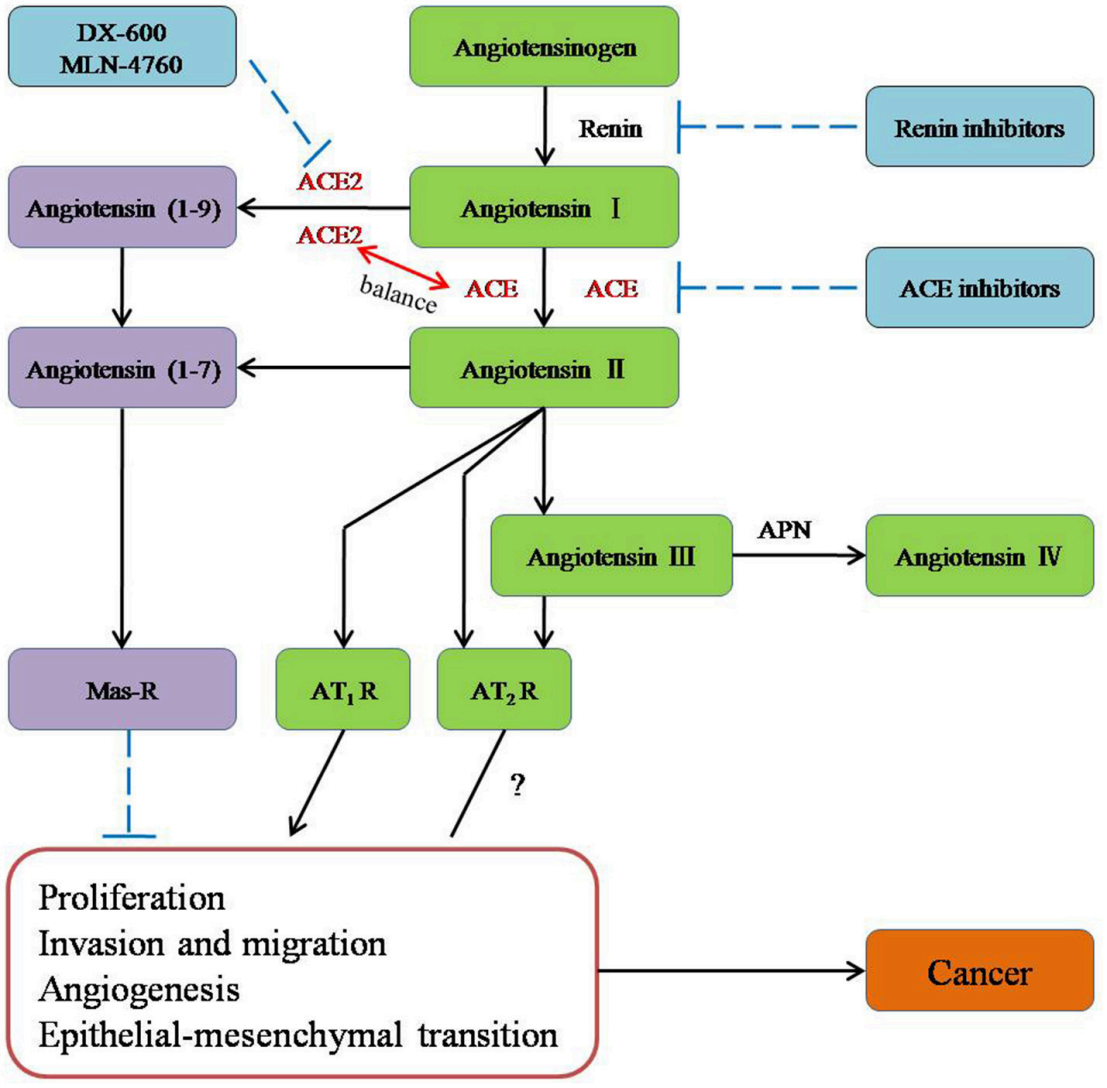
**The role of new members of the RAS system in cancer and potential molecules for targeting the RAS system in therapeutic application**.

The conventional RAS, which consists of renin, ACE, angiotensinogen, Ang I, Ang II, AT1R, AngII type 2 receptor (AT2R), and chymase, is considered a cascade that leads to the conversion of the inactive pro-hormone. The classical RAS focuses on ACE, Ang II, AT1R, and the interactions among them (Figure 1).

ACE is a key enzyme of the RAS, and it plays a central role in the generation of the active peptide hormone of Ang II from Ang I via cleavage. Although Ang II is recognized as a potent mitogen, it is also a major regulator of cardiovascular homeostasis and blood pressure and the essential biologically active peptide of the RAS. Recently, it has been shown that Ang II is involved in the regulation of cell proliferation, inflammation, migration, and tissue remodeling as well as angiogenesis. Additionally, as a receptor of Ang II, AT1R is involved in breast cancer (Zhao et al., 2010) and ovarian carcinoma (Suganuma et al., 2005). Furthermore, researchers have suggested that the ACE-Ang II-AT1R pathway is related to the biology process leading to cancer (Okamoto et al., 2010; Zhao et al., 2010; Gallagher et al., 2011; Rodrigues-Ferreira et al., 2012).

ACE2 [also known as ACE-related carboxypeptidase or angiotensin-converting enzyme homolog (ACEH)] is mainly expressed in the renal tubular epithelium and vascular endothelial cells. Moreover, ACE2 is known as a homolog of ACE; it presents 40% identity and 61% similarity to ACE and is an 805-amino-acid type-I trans-membrane protein that contains an extracellular (ecto) domain (amino acids 18–739), a trans-membrane region (amino acids 740–768), and an intracellular tail (Donoghue et al., 2000; Tipnis et al., 2000). ACE2 mainly cleaves Ang II to Ang-(1–7), whereas ACE activity primarily generates Ang II by cleaving Ang-(1–9) (Jia, 2016). The axis formed by ACE2 is a potent counter-regulator against ACE activity and plays a protective role against many diseases, especially carcinoma (Han and Ge, 2016). As part of the axis formed by ACE2, Ang-(1–7) is an endogenous heptapeptide hormone that mediates biological activity through Mas, whose production has been found to be dysregulated in certain cancers, such as breast cancer (Luo et al., 2015), lung cancer, and prostate cancer (Gallagher and Tallant, 2004; Krishnan et al., 2013a).

Drugs play a very important role in the treatment of cancer, and the RAS has been shown to play a special role in the occurrence of cancer drug resistance.

Previous studies have reported that prostatic RAS components are overexpressed in hormone refractory prostate cancer tissue, and the expression of these components is influenced by several types of hormonal stimulation (Uemura et al., 2006). Research has also found that RAS could influence the immune response, which may be potentially useful in cancer treatments. A study also reported that the blockade of ACE or the AT1 receptor may reduce tumor growth (Shen et al., 2007). Ager et al. (2011) reported that the blockade of the classical RAS through AT1R blockade or ACE inhibition reduces tumor growth in several experimental mouse models of cancer. Conversely, the activation of the alternative RAS, through Ang-(1–7) infusion or AT2R activation, can also reduce tumor growth. Cheng et al. (2016) found that ACE2 overexpression may potentially suppress angiogenesis in non-small cell lung cancer (NSCLC) after the development of acquired platinum resistance. Namazi et al. (2014) suggested that the combination of either captopril or captopril+losartan with innate and acquired tamoxifen resistance led to the prevention and even reversion of innate and acquired tamoxifen resistant phenotype.

Additionally, a study in bladder cancer has found that the ACE-Ang II-AT1 receptor axis in the local RAS promotes VEGF production in platinum-resistant tumors (Tanaka et al., 2011), whereas the RAS new branch ACE2-Ang-(1–7)-Mas axis could reduce the production of VEGF in drug-resistant tumors, thereby inhibiting angiogenesis, although the reversal of tumor resistance has not yet been reported.

### ACE2/Ang-(1–7)/MasR in cancer

#### Role of the ACE2/Ang-(1–7)/MasR axis in cancer

Many components of the RAS are expressed in various cancers, including breast (Luo et al., 2015), gastric (Carl-McGrath et al., 2007), colon (Bernardi et al., 2012), and laryngeal (Han and Ge, 2016) cancers. The ACE2/Ang-(1–7)/MasR axis, which represents a newly discovered component of the RAS, has been shown to be up-regulated or down-regulated in different cancers. Yu et al. (2016) suggested that ACE2 expression is decreased in breast cancer, NSCLC (Feng et al., 2010), hepatocellular carcinoma (Ye et al., 2015), and pancreatic cancer (Zhou et al., 2009), and Zong et al. (2015) reported that ACE2 levels are lower in gallbladder cancer cells than in normal gallbladder cells. Ang-(1–7) is generated primarily from Ang I or AngII via enzymatic cleavage; in recent studies, ACE2 has been identified as the main enzyme that generates Ang-(1–7), whereas ACE has been shown to be responsible for cleaving Ang-(1–7) to produce Ang-(1–5). Luo et al. found that the MasR is a receptor for Ang-(1–7), which is derived from Ang II via the action of ACE2 and is reduced in breast cancer (Luo et al., 2015). The MasR has been found to be significantly up-regulated in colon cancer tissues (Bernardi et al., 2012) and in association with colorectal cancer metastasis (Neo et al., 2010) compared with levels in non-neoplastic colon mucosal tissue.

Thus, different assays present different results for ACE2/Ang-(1–7)/MasR expression, as illustrated in Table 1. The reason for these different outcomes may be related to the low expression levels of the proteins, as well as the low selectivity and sensitivity of the antibodies used in the assays, which increases the difficulty in precisely measuring expression levels. Thus, for experiments focused on ACE2/Ang-(1–7)/MasR axis components, more than one immune-based detection method should be used, and mRNA analyses are preferred.

**Table 1:** **ACE2/Ang1-7/Mas receptor axis and modulation in previous studies**.

**Organ/model**	**Compounds or strategy used**	**Effect**	**References**
Breast cancer	ACE2 overexpression	↓ Store operated calcium entry	Yu et al., 2016
		↓ Pak1/NF-κB/snail 1 pathways	
		↑E-cadherins	
Hepatocellular carcinoma	ACE2 overexpression	↓ Serum concentrations of Ang II	Ye et al., 2015
		↓ Ang-(1–7) and VEGF	
		↓ Hepatic mRNA levels of CD34	
		↓ Grade of disease severity	
Gallbladder cancer	ACE2 supplement	↓ Tumor cell growth	Zong et al., 2015
		↑ ERK	
Lung carcinoma	ACE2 overexpression	↓ VEGF expression	Fan et al., 2014
Osteosarcoma	ACE2 upregulated	↓ Tumor growth and metastasis	Ender et al., 2014
Laryngeal cancer	ACE2 overexpression	↑ Prognosis	Fountzilas et al., 2013
Gallbladder cancer	ACE2 overexpression	↓ Tumor size	Li et al., 2014
		↓ TNM stage	
		↓ Lymph node metastasis	
Lung cancer	ACE2 overexpression	↓ Metastasis	Qian et al., 2013
		↓ EMT	
Lung cancer	ACE2 overexpression	↓ Invasion and angiogenesis	Feng et al., 2010
Pancreatic cancer	ACE2 overexpression	↓ Proliferation and tumorigenicity	Zhou et al., 2011
		↓ VEGFa	
Lung cancer	ACE2 overexpression	↓ Growth and VEGFa	Feng et al., 2010
Pancreatic cancer	ACE2 downregulation	↑ Proliferation	Zhou et al., 2009
Lung cancer	Ang-(1–7) overexpression	↓Cdc6	Chen et al., 2016
		↓Angiogenesis	
Lung cancer	Ang-(1–7)	↓miRNA-149-3p	Silva Bde et al., 2016
		↓ Migration	
Nasopharyngeal carcinoma	Ang-(1–7) overexpression	↓ Proliferation	Pei et al., 2016
		↓ Migration	
		↓ Vessel density	
Hepatocellular carcinoma	Ang-(1–7) overexpression	↓ Tumor cell proliferation	Liu et al., 2015
		↑Tumor apoptopsis	
Renal carcinoma	Ang-(1–7) overexpression	↑ Migration and invasion	Zheng et al., 2015
Prostate cancer	Ang-(1–7) overexpression	↓ Metastasis and proliferation	Krishnan et al., 2013a
Breast cancer	Ang-(1–7)	↓ Cancer cell growth	Luo et al., 2015
		↓ Anti-apoptotic survival	
		↓ Invasion	
Lung cancer	Ang-(1–7) overexpression	↓ Angiogenesis	Soto-Pantoja et al., 2009

#### ACE2/Ang-(1–7)/MasR: novel biomarkers for cancer?

As previously mentioned, low ACE2 activity levels are frequently associated with the presence of cancer. Additionally, increased serum ACE2 activity has been reported in healthy individuals (Zong et al., 2015). Moreover, researchers have suggested that decreased ACE2 activity may reflect the presence of cancer associated with diabetes (Pedersen et al., 2015). Yu et al. (2016) reported low ACE2 expression levels in breast cancer samples with distant metastasis and in samples of tumors that had spread to lymph nodes. Ye et al. reported that patients with higher levels of ACE2 expression had longer survival times than those with a lower levels of ACE2 expression, which suggests that a low level of ACE2 expression may be a useful indicator of poor prognoses for patients with hepatocellular carcinoma (Ye et al., 2015). The expression levels of ACE2 in different cancers are shown in Table 1, and the results suggest that measurements of ACE2 activity maybe more valuable for predicting the occurrence of adverse events.

Evidence has shown that ACE2 is a prognostic biomarker in gallbladder carcinoma and is involved in tumor growth, angiogenesis, metastasis, and invasion in lung cancer. Additionally, Ang-(1–7) regulates the migration and invasion of carcinoma cells via Mas, and the MasR might act as an inhibitory regulator of breast cancer. Additional cancer types, such as hepatocellular carcinoma, colon cancer, and laryngeal cancer, have demonstrated an association with the ACE2/Ang-(1–7)/MasR axis.

Together, these studies suggest that measuring ACE2 activity may be a helpful diagnostic and prognostic tool for indicating patients with cancer. Although whether the origin of soluble ACE2 is from increased tissue synthesis or augmented tissue shedding remains unknown, it may reflect a compensatory but insufficient response to adverse stimuli.

#### Pleiotropic roles and mechanisms of the ACE2/Ang-(1–7)/MasR axis in cancer

As previously mentioned, the components of the ACE2/Ang-(1–7)/MasR axis have various functions in different cancer types, and Yu et al. suggested that the down-regulation of the ACE2/Ang-(1–7)/MasR axis could promote the metastasis of breast cancer (Yu et al., 2016). Zhou et al. reported that the loss of ACE2 expression promotes the development of gallbladder cancer (Zong et al., 2015). Zhou et al. suggested that the expression of ACE2 was decreased in pancreatic ductal adenocarcinoma tissues in which Ang II had accumulated (Zhou et al., 2009). Compared with these anti-cancer roles, the ACE2/Ang-(1–7)/Mas axis has been shown to promote the migration and invasion of renal cell carcinoma (Zheng et al., 2015) and mediate the AngII-induced epithelial-mesenchymal transition (EMT) in tubule cells (Burns et al., 2010). The mechanisms that generate these contradictory effects of the ACE2/Ang-(1–7)/MasR axis on cancer require additional investigation.

The mechanisms regulating cancer include the following aspects.

##### Cell proliferation

Yu et al. (2016) reported that the RAS is an important component of the tumor microenvironment and plays a key role in promoting cancer cell proliferation, metabolism, migration, and invasion, as well as angiogenesis. These authors observed that the branch of the ACE2/Ang-(1–7)/MasR axis connected to the RAS is associated with anti-proliferative and anti-metastatic properties, and they further found that the ACE2 protein levels are negatively correlated with the metastatic ability of breast cancer cells and the grade of breast tumors and showed that the up-regulation of the ACE2/Ang-(1–7)/MasR axis could inhibit breast cancer cell metastasis *in vivo* and *in vitro* (Yu et al., 2016). Another research group revealed that reduced ACE2 expression via RNA interference promotes the proliferation of cultured pancreatic cancer cells, suggesting that the inhibition of ACE2 may have clinical potential as a novel molecular target for the treatment of pancreatic ductal adenocarcinoma and the reduction of cell proliferation (Zhou et al., 2009, 2011).

A human lung tumor xenograft model showed that Ang-(1–7) treatment reduces tumor volume in mice and inhibits cell proliferation via the reduction of COX-2 activity (Menon et al., 2007). Other investigations using human nasopharyngeal xenografts have revealed that Ang-(1–7) inhibits tumor growth via anti-angiogenic activities (Pei et al., 2016).

Mas1 is regarded as an oncogene, and it encodes the receptor for Ang-(1–7). Luo et al. (2015) found that Mas expression levels were inversely associated with the proliferation index of invasive ductal carcinoma of the breast tissue.

**Figure 2:**
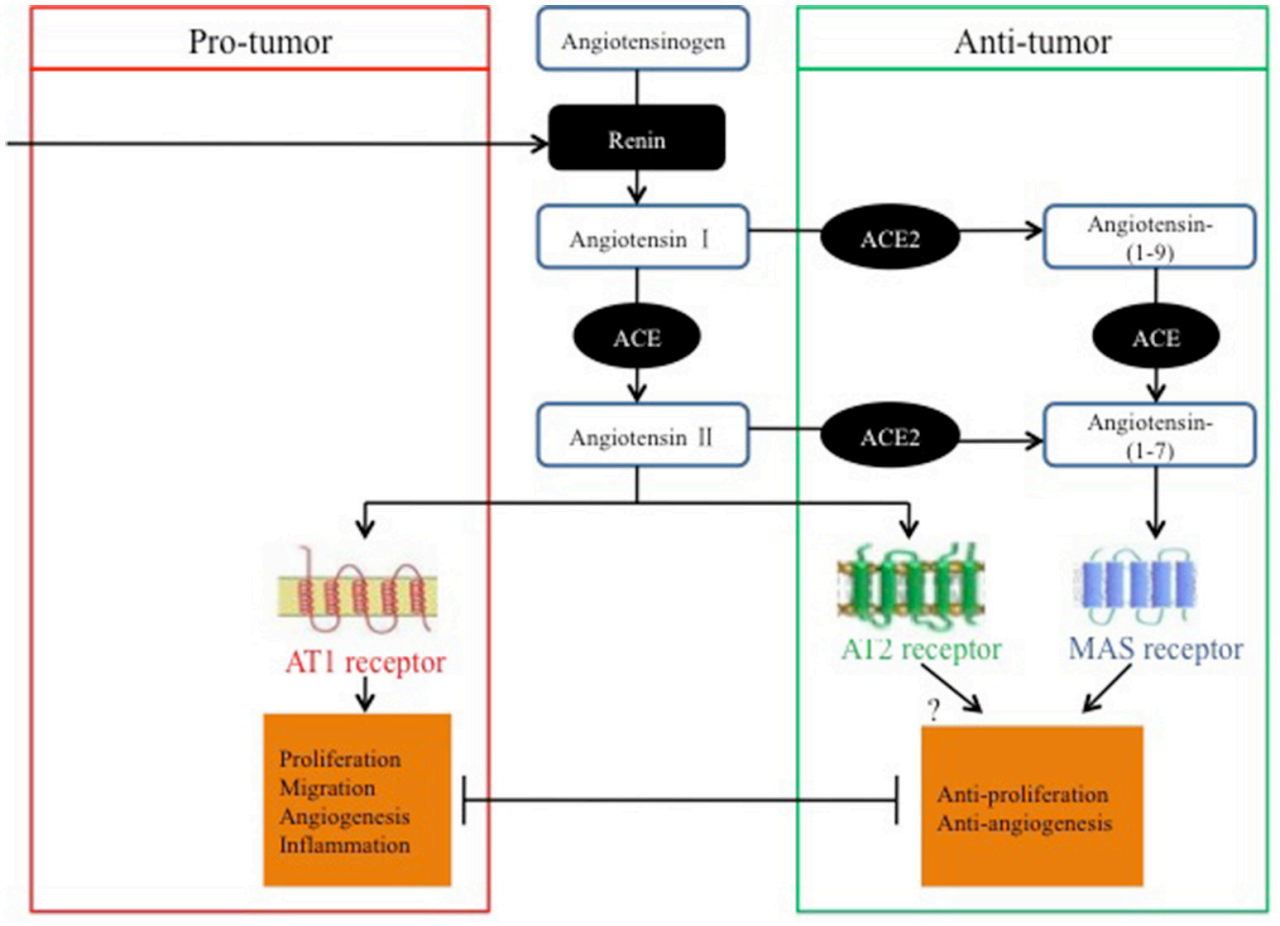
**Pro-tumor and anti-tumor balance of the RAS in relation to classical and alternative pathways**.

Ender et al. (2014) discovered that the knockdown of Mas expression mediated by small interfering RNA leads to increased cell proliferation in osteosarcoma and suggested that targeting the ACE2/Ang-(1–7)/Mas axis may be beneficial for the treatment of osteosarcoma by reducing cancer cell proliferation and preventing cancer metastasis (Figure 2).

##### Invasion and migration

Excess extracellular matrix (ECM) degradation is one of the hallmarks of tumor invasion and migration (Huang et al., 2005). Matrix metalloproteinases (MMPs) are a large family of at least 20 zinc-dependent neutral endopeptidases that can collectively degrade all known components of the ECM. Among the human MMPs, MMP-2 and MMP-9 show substrate specificity toward type IV collagen, the major component of the basement membrane. The expression of these two MMPs is strongly linked to tumor metastasis in various types of human cancer (Mook et al., 2004).

Feng et al. (2011) found that ACE2 overexpression inhibits tumor invasion, metastasis, and MMP production, suggesting that ACE2 overexpression suppresses the invasion and migration of NSCLC cells, which may occur by decreasing MMP-2 and MMP-9 activity.

MMP expression is regulated by PI3K/Akt, P38, and MAPK and it is known as a mediator of lung cancer metastasis. Ang-(1–7) has been identified as an inhibitor of A549 human lung adenocarcinoma cells that acts via the inactivation of the PI3K/Akt, P38, and MAPK signaling pathways (Ni et al., 2012). The NF-κB and PAK signaling pathways have been associated with aggressive cancer. The up-regulation of the ACE2/Ang-(1–7)/MasR axis promotes the expression of E-cadherin by suppressing the PAK1/NF-κB/Snail1 pathway, and the activatedACE2/Ang-(1–7)/MasR axis inhibits breast cancer metastasis and store-operated calcium entry (SOCE). However, SOCE participates in breast cancer migration and the NF-κB and PAK signaling pathways, and the down-regulation of the ACE2/Ang-(1–7)/MasR axis inhibits breast cancer metastasis by enhancing SOCE (Yu et al., 2016).

In prostate cancer, investigators exploring the relationship between Ang-(1–7) and prostate cancer metastasis found an association between Ang-(1–7) and vascular endothelial growth factor (VEGF) and determined that Ang-(1–7) reduces metastasis via anti-angiogeneic activities (Krishnan et al., 2013b). However, in renal cell carcinoma, Ang-(1–7) promoted migration and invasion in a manner dependent on MasR-induced Akt activation (Zheng et al., 2015). These discrepancies might be related to the different detection methods used in these studies, different signaling pathways, and different types of cancer.

##### Promotion of tumor-associated angiogenesis

VEGFa is an important mediator of angiogenesis. Feng et al. (2010) found that VEGFa protein expression and mRNA production in A549 cells are increased via stimulation with 10 μM AngII, which suggests that the RAS in tumors promotes tumor angiogenesis via VEGFa induction. These researchers also found that VEGFa expression was decreased in the supernatants of A549 cells infected with murine stem cell virus (MSCV)-ACE2 compared with expression in cells infected with the vector alone (Feng et al., 2010). These findings indicate that ACE2 may inhibit tumor growth by decreasing angiogenesis in lung cancer.

In further studies, Feng et al. (2011) confirmed that ACE2 overexpression inhibits cell growth and VEGFa production while simultaneously suppressing ACE and Ang II expression in human lung cancer xenografts, and these findings suggest that ACE2 overexpression may suppress the invasion and angiogenesis in NSCLC.

Ang-(1–7) anti-angiogenesis activities may function via the attenuation of VEGF and VEGF receptors in nasopharyngeal carcinoma (Pei et al., 2016) and in lung cancer (Soto-Pantoja et al., 2009).

##### Induction of the epithelial-mesenchymal transition

The EMT plays a fundamental role in tumor progression and the formation of metastases. In the EMT, epithelial tumor cells with a cobblestone phenotype acquire mesenchymal cell characteristics, such as a spindle/fibroblast-like morphology. This process involves the loss or down-regulation of epithelial markers, including E-cadherin, and the up-regulation of mesenchymal molecular markers, such as vimentin and α-smooth muscle actin (α-SMA). During the EMT, the loss of epithelial markers, especially E-cadherin, is a critical process that is regulated by several important transcriptional repressors. The EMT may be triggered by many growth factors, including transforming growth factor β1 (TGF-β1), which is the most important factor that can be influenced by the tumor microenvironment.

Qian et al. (2013) reported that ACE2 up-regulates the expression of E-cadherin both *in vitro* and *in vivo* and that it down-regulates vimentin, which are both representative markers of the EMT. Furthermore, a western blot analysis indicated that ACE2 attenuates the TGF-β1-mediated EMT of A549 cells. ACE2 has been found to decrease the transcriptional levels of genes associated with the EMT *in vitro*, and exposing cells to DX600, an inhibitor of ACE2, recovers the sensitivity of lung cancer cells to TGF-β1 (Qian et al., 2013). These data suggest that ACE2 attenuates lung cancer metastasis by inhibiting the EMT, and they further indicate that ACE2 may represent a potential therapeutic target in treating lung cancer, where the EMT contributes to the development of tumor metastasis.

## Conclusions

In summary, most studies have suggested that the ACE2/Ang-(1–7)/MasR axis has anti-tumor properties that may be exerted via pathways involved in anti-proliferation, invasion and migration suppression, tumor-associated angiogenesis, and the EMT; however, several studies have proposed contradictory effects. As previously stated, the evidence for opposing roles of the ACE2/Ang-(1–7)/MasR axis in cancer might be dependent on the cancer type and on variations in the experimental methods used.

The exact mechanisms underlying the contributions of the different components of the ACE2/Ang-(1–7)/MasR axis to cancer progression require further investigation, and the therapeutic potential of the different components remains controversial.

Otherwise, it has been reported that angiotensin I-converting enzyme inhibitors (ACEI) could decrease tumor growth and tumor-associated angiogenesis and inhibit metastasis. Miao et al. reported that ACEI in combination with standard chemotherapy or TKIs had a positive effect on progression-free survival after first-line therapy or overall survival, regardless of whether the lung cancer was in the early or advanced stage (Miao et al., 2016). Wilop et al. concluded that addition of ACEI to platinum-based first-line chemotherapy may contribute to prolonged survival in patients with advanced lung cancer (Wilop et al., 2009). However, Raimondi et al. suggested that no association of ACEI use with disease free and overall survival was found (Raimondi et al., 2016). Therefore, relationship between ACEI and tumor needs more research to confirm.

Thus, based on the above review, the following questions require further study: What is the effect of the microenvironment (e.g., glucose metabolism, fat metabolism, cholesterol metabolism, and inflammation) on balancing the ACE2/Ang-(1–7)/MasR axis and how can this environment be properly regulated? How does the ACE2/Ang-(1–7)/MasR balance switch to exert an anti-tumor effects, and what is the exact role of the axis in the development of cancer? Should more reliable approaches be developed to prevent or reverse cancer using agonists or antagonists of these newly discovered RAS members? Developing a greater understanding of these issues will provide additional strategies for cancer intervention.

## Author contributions

All authors participated in literature research and data classification. JX, JF, and FW wrote the manuscript. YJ reviewed and edited the manuscript before submission.

### Conflict of interest statement

The authors declare that the research was conducted in the absence of any commercial or financial relationships that could be construed as a potential conflict of interest.

## Abbreviations

ACE2: angiotensin-converting enzyme 2
RAS: renin-angiotensin system
Ang II: angiotensin II
Ang-(1–7): angiotensin-(1–7)
AT1R: angiotensin II type 1 receptor
AT2R: angiotensin II type 2 receptor
ECM: extracellular matrix
MMPs: matrix mettaloproteinases
VEGF: vascular endothelial growth factor
MSCV: murine stem cell virus
EMT: epithelial mesenchymal transition
α-SMA: α-smooth muscle actin
TGF-β: transforming growth factor-β
ACEI: angiotensin-converting enzyme inhibitors
ARB: angiotensin II receptor antagonists.
